# Supplementary material for: Functional profiling of single CRISPR/Cas9-edited human long-term hematopoietic stem cells
Source: Nat Commun. 2019 Oct 18;10:4730. doi: 10.1038/s41467-019-12726-0 (PMC6802205; doi:10.1038/s41467-019-12726-0)
Supplement: Supplementary file 1 — Supplementary Information [file 41467_2019_12726_MOESM1_ESM.pdf]

Functional Profiling of Single CRISPR/Cas9-Edited Human Long-Term Hematopoietic Stem Cells

Supplementary Information

Wagenblast et al.

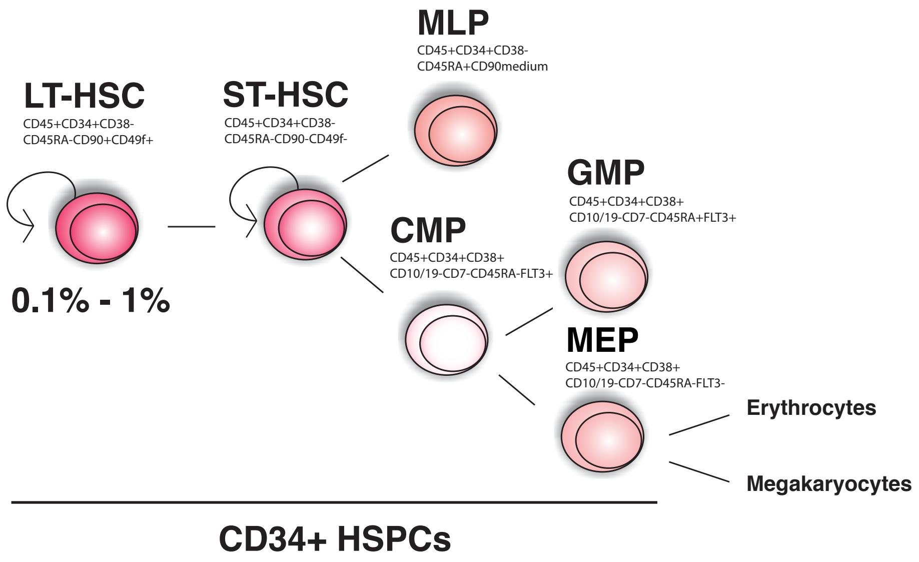

Supplementary Figure 1: Lineage hierarchy of human hematopoietic stem cells. Major classes of stem and progenitor cells, which are defined by cell surface markers. Long-term hematopoietic stem cells (LT-HSCs), which are defined by the expression of CD49f and other markers, constitute to only about 0.1 - 1% of bulk CD34+ hematopoietic stem and progenitor cells (HSPCs). LT-HSCs give rise to various types of progenitor cells including short-term hematopoietic stem cells (ST-HSCs), multi-lymphoid progenitor cells (MLPs), common myeloid progenitor cells (CMPs), granulocyte/macrophage progenitor cells (GMPs) and myelo–erythroid progenitor cells (MEPs). MEPs terminally differentiate into erythrocytes and megakaryocytes.

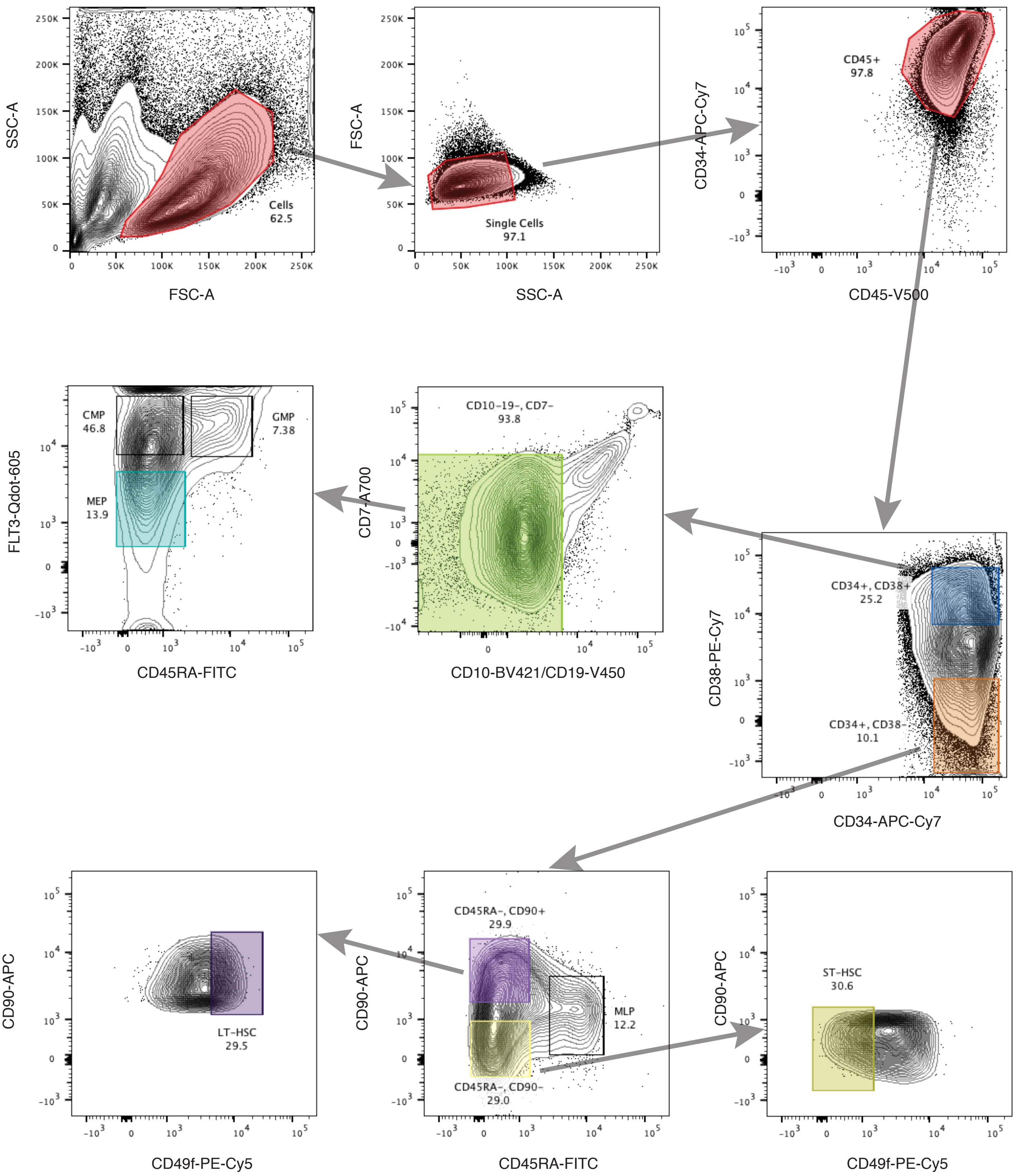

Supplementary Figure 2: Flow cytometry sorting scheme. Lineage depleted neonatal cord blood was utilized to sort LT-HSCs as CD45+CD34+CD38–CD45RA–CD90+CD49f+, ST-HSCs as CD45+CD34+CD38–CD45RA–CD90–CD49f– and MEPs as CD45+CD34+CD38+CD10/19–CD7–CD45RA–FLT3–.

Wagenblast et al. Supplementary Figure 3

a

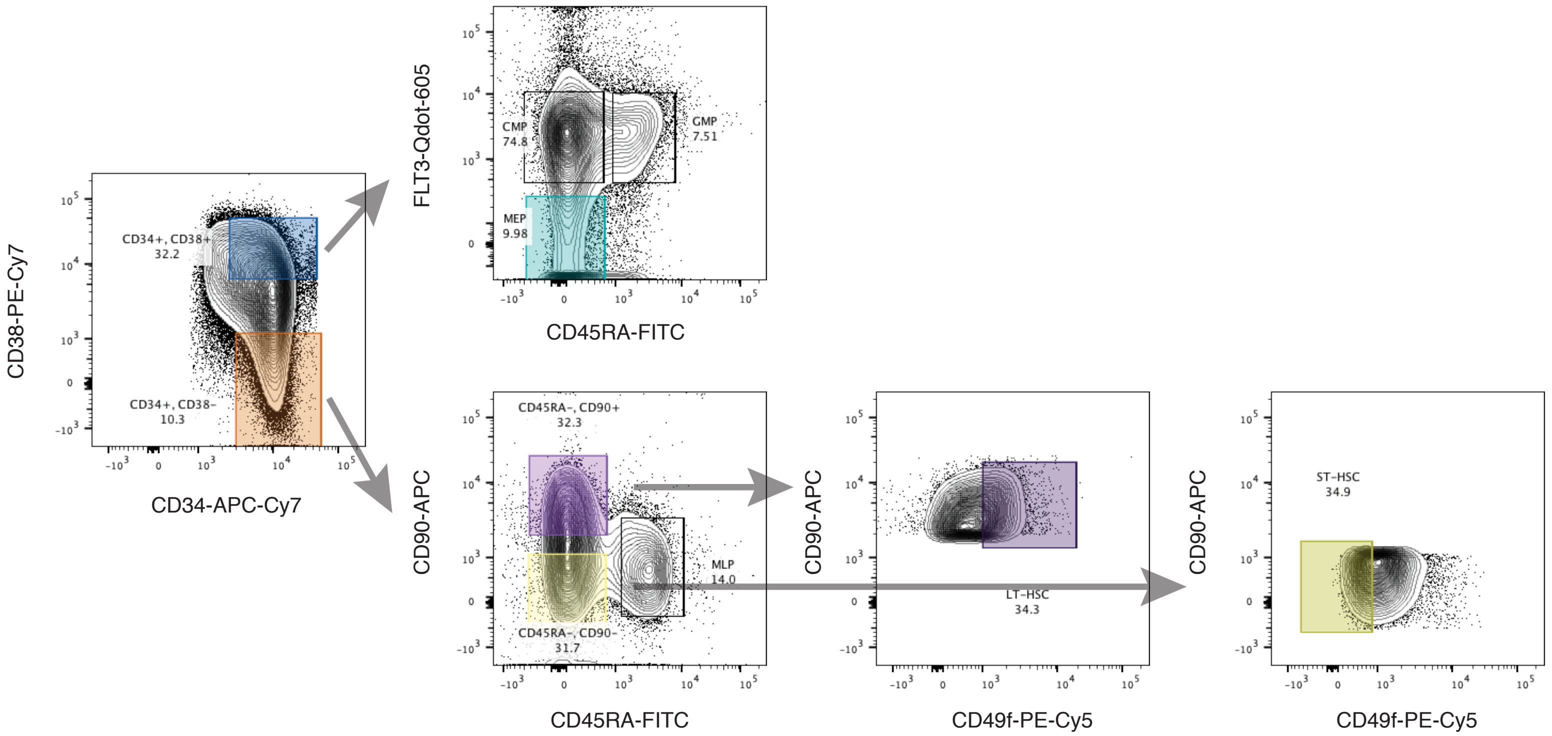

b

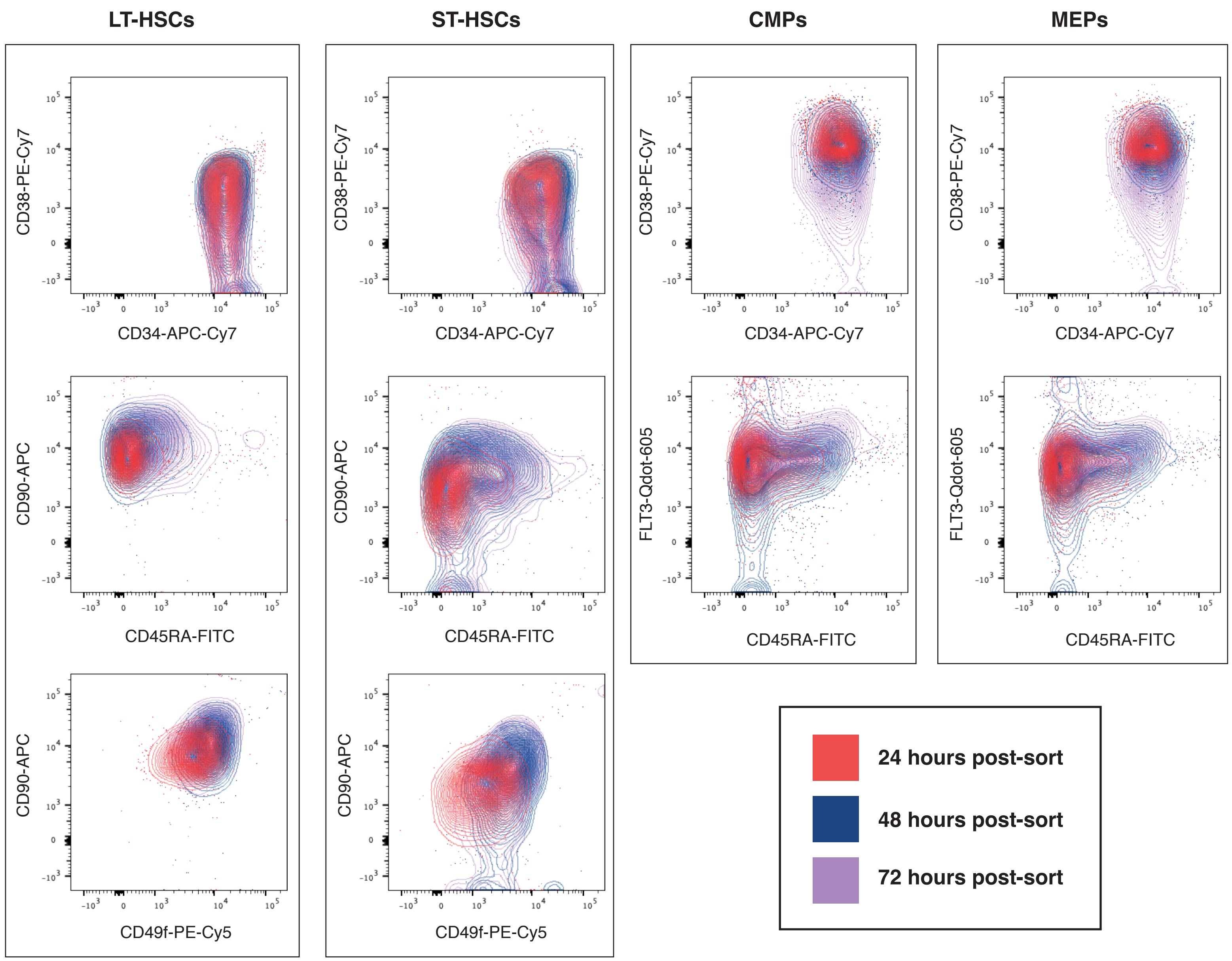

Supplementary Figure 3: Analysis of immunophenotypic cell surface markers after in vitro culture. **a** Lineage depleted cord blood was sorted into LT-HSCs (CD45+CD34+CD38–CD45RA–CD90+CD49f+), ST-HSCs (CD45+CD34+CD38–CD45RA–CD90–CD49f–), CMPs (CD45+CD34+CD38+CD10/19–CD7–CD45RA–FLT3+) and MEPs (CD45+CD34+CD38+CD10/19–CD7–CD45RA–FLT3–) and cells were cultured for 24, 48 and 72 hours. **b** Immunophenotype of previously sorted LT-HSCs, ST-HSCs, CMPs and MEPs after 24, 48 and 72 hours in culture.

Wagenblast et al. Supplementary Figure 4

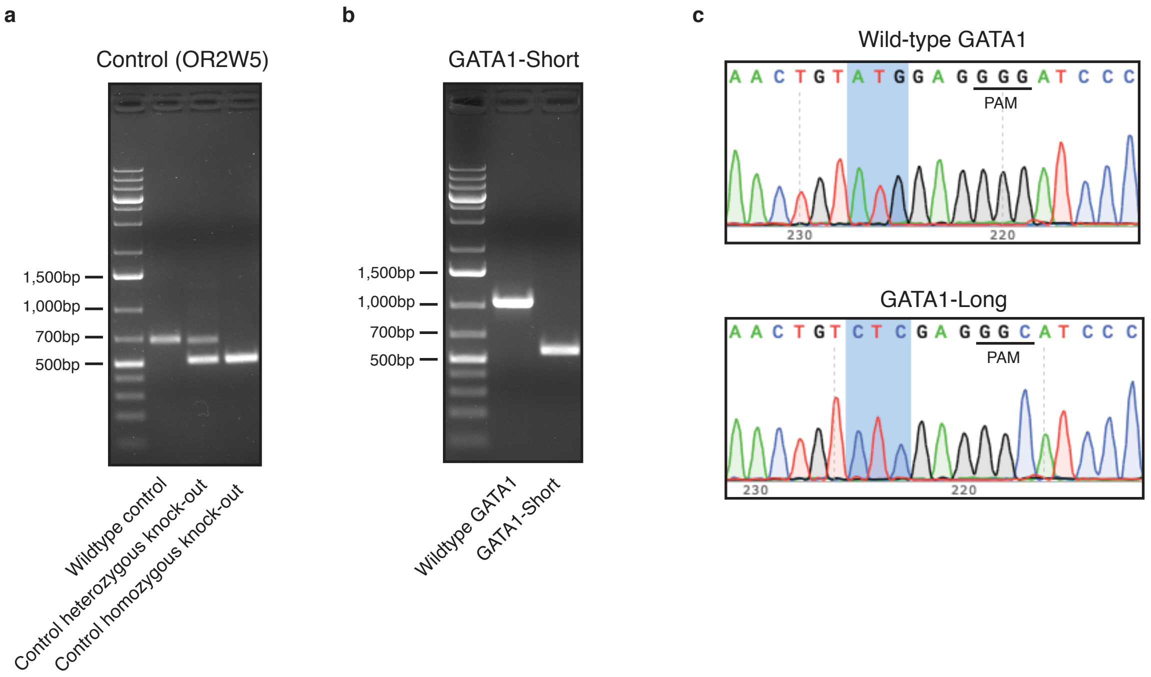

Supplementary Figure 4: CRISPR/Cas9-mediated isoform assignment to GATA1-Short and –Long. a Gel electrophoresis analysis of single cell-derived colonies that were CRISPR/Cas9 edited with control gRNAs. Only homozygous knock-out colonies were utilized in the analysis. **b** Gel electrophoresis analysis of single cell-derived colonies that were CRISPR/Cas9 edited for GATA1-Short. GATA1 is located on the X-chromosome and only male cord blood samples were utilized. **c** Sanger sequencing analysis of single cell-derived colonies that were CRISPR/Cas9 edited for GATA1-Long. The alternative start site was mutated from ATG to CTC and the PAM sequence mutated from GGG to GGC to avoid repeated cutting by the gRNA.

a

		# cells sorted	# colonies	# of CRISPR-edited colonies
LT-HSC	Control	341	147	81
	GATA1-Long	280	130	24
	GATA1-Short	226	98	36
ST-HSC	Control	311	233	145
	GATA1-Long	327	243	69
	GATA1-Short	274	188	68
MEP	Control	273	164	90
	GATA1-Long	315	187	58
	GATA1-Short	289	167	65

b

gRNA	# of mismatches	Chromosome location [hg38]	Cleavage efficiency [measured by TIDE]
GATA1-Short gRNA-1	2	chr10_20221643	no cleavage
GATA1-Short gRNA-1	2	chr18_8376641	no cleavage
GATA1-Short gRNA-1	3	chr1_223704955	no cleavage
GATA1-Short gRNA-1	3	chr4_118059061	no cleavage
GATA1-Short gRNA-1	3	chr10_53712490	no cleavage
GATA1-Short gRNA-1	3	chr14_72742463	no cleavage
GATA1-Short gRNA-1	3	chr15_52109094	no cleavage
GATA1-Short gRNA-1	3	chr16_35544187	no cleavage
GATA1-Short gRNA-1	3	chr16_71816748	no cleavage
GATA1-Short gRNA-1	3	chr17_80332977	no cleavage
GATA1-Short gRNA-1	3	chr19_32673314	no cleavage
GATA1-Short gRNA-1	3	chr22_44756609	no cleavage
GATA1-Short gRNA-2	3	chr3_127784219	no cleavage
GATA1-Short gRNA-2	3	chr5_178537945	no cleavage
GATA1-Short gRNA-2	3	chr7_17679912	no cleavage
GATA1-Short gRNA-2	3	chr8_107174119	no cleavage
GATA1-Short gRNA-2	3	chr10_123397080	no cleavage
GATA1-Short gRNA-2	3	chr12_2085541	no cleavage
GATA1-Short gRNA-2	3	chr13_113094529	no cleavage
GATA1-Short gRNA-2	3	chr21_42868084	no cleavage
GATA1-Short gRNA-2	4	chr8_35663175	no cleavage
GATA1-Long gRNA-1	3	chr2_15662510	no cleavage
GATA1-Long gRNA-1	3	chr9_76619884	no cleavage
GATA1-Long gRNA-1	3	chr12_20017977	no cleavage
GATA1-Long gRNA-1	3	chr12_94844478	no cleavage
GATA1-Long gRNA-1	4	chr3_77895170	no cleavage
GATA1-Long gRNA-1	4	chr4_22175316	no cleavage
GATA1-Long gRNA-1	4	chr7_40859472	no cleavage
GATA1-Long gRNA-1	4	chr8_109403836	no cleavage
GATA1-Long gRNA-1	4	chr8_81290900	no cleavage
GATA1-Long gRNA-1	4	chr13_51458879	no cleavage
GATA1-Long gRNA-1	4	chr15_62326516	no cleavage
Control gRNA-1	2	chr2_30448683	no cleavage
Control gRNA-1	2	chr6_169886852	no cleavage
Control gRNA-1	2	chr20_52425093	no cleavage
Control gRNA-1	3	chr7_3081168	no cleavage
Control gRNA-1	3	chr7_135691300	no cleavage
Control gRNA-1	4	chr1_191051674	no cleavage
Control gRNA-1	4	chr3_55088990	no cleavage
Control gRNA-1	4	chr5_4882788	no cleavage
Control gRNA-1	4	chr12_114950867	no cleavage
Control gRNA-1	4	chr16_48579049	no cleavage
Control gRNA-1	4	chr21_34622361	no cleavage
Control gRNA-2	3	chr15_54082949	no cleavage
Control gRNA-2	4	chr2_118858930	no cleavage
Control gRNA-2	4	chr3_122079794	no cleavage
Control gRNA-2	4	chr4_1100146	no cleavage
Control gRNA-2	4	chr5_2514714	no cleavage
Control gRNA-2	4	chr7_2644811	no cleavage
Control gRNA-2	4	chr8_10811909	no cleavage
Control gRNA-2	4	chr12_12496109	no cleavage
Control gRNA-2	4	chr14_96046026	no cleavage
Control gRNA-2	4	chr16_85653916	no cleavage
Control gRNA-2	4	chr19_18226209	no cleavage
Control gRNA-2	4	chr21_42232307	no cleavage

c

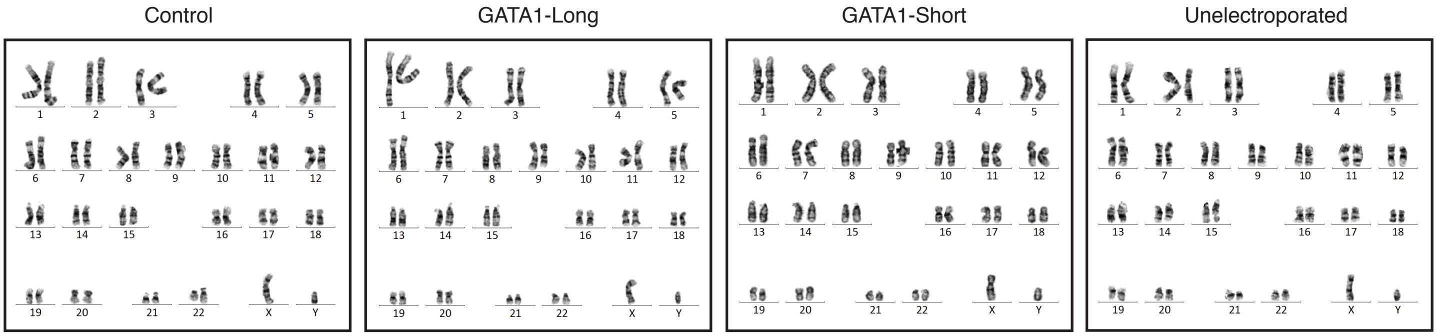

Supplementary Figure 5: Off-target analysis of CRISPR/Cas9. a Total number of single cells sorted, number of single cell derived colonies and number of CRISPR/Cas9-edited colonies for each cell type for the in vitro differentiation assay (n = 3 experiments with independent cord blood pools). **b** Off-target cleavage efficiency of genomic loci that were similar in sequence to the gRNA target sequence (n = 20-30 single cell-derived colonies per genomic loci). **c** G-banding karyotype analysis on control, GATA1-Long and –Short CRISPR/Cas9-edited cells (n = 20 metaphases per condition, shown one)

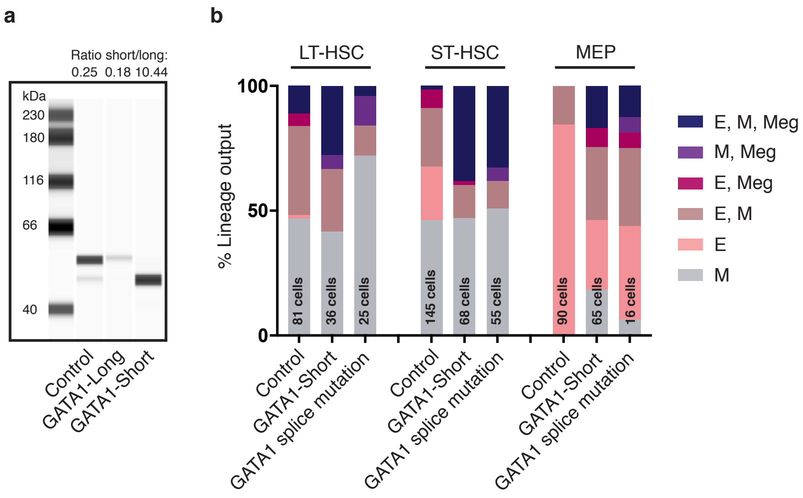

c

		E	M	Meg
LT-HSC	Control	53.10	98.80	16.04
	GATA1-Short	52.80	100.00	33.30
	GATA1-Short splice mutation	16.00	100.00	16.00
ST-HSC	Control	53.79	71.00	8.97
	GATA1-Short	52.93	98.53	39.70
	GATA1-Short splice mutation	43.60	100.00	38.17
MEP	Control	100.00	15.60	0.00
	GATA1-Short	81.55	64.61	24.61
	GATA1-Short splice mutation	87.50	52.25	25.00

d

	Sequence of GATA1 5' exon 2 splice site	Frequency
Wildtype	5'-CCAGGTAAC-3'	
Splice mutants	5'-ATTGAGTGG-3'	57/96
	5'-CCAGTAACT-3'	37/96
	5'-CCAGTGGGA-3'	1/96
	5'-CCATTCCAA-3'	1/96

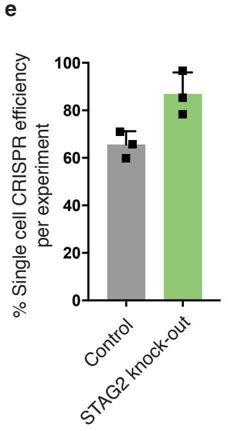

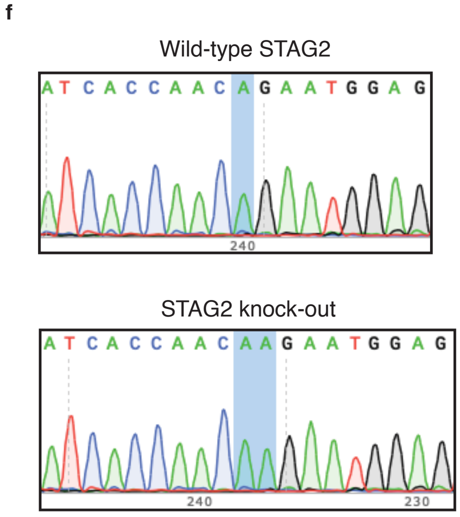

Supplementary Figure 6: Single cell in vitro differentiation assay. a Capillary-based western assay of GATA1 in bulk MEPs that were CRISPR/Cas9 edited with control, GATA1-Long and GATA1-Short gRNAs and cultured under erythro-myeloid conditions for three days (n = 2 experiments, second western assay is shown in Fig. 2c). **b** Lineage output from the in vitro single cell differentiation assay of individual CRISPR/Cas9 edited LT-HSCs, ST-HSCs and MEPs with control, GATA1-Short and GATA1 Exon 2 splice mutation. Numbers of single cell colonies with positive genotype are indicated at each condition (E = erythroid, M = myeloid, Meg = megakaryocytic, n = 3 experiments with independent cord blood pools, number of single cells assessed in each condition is indicated in each bar graph, unpaired t-test P < 0.01 for E, M, Meg in GATA1 splice mutation versus Control among ST-HSCs and MEPs). **c** Overall percentage of erythroid, myeloid and megakaryocyte containing colonies from single cell in vitro differentiation assay with GATA1 Exon 2 splice mutations (E = erythroid, M = myeloid, Meg = megakaryocytic, n = 3 experiments with independent cord blood pools, unpaired t-test P < 0.05 for % Meg in GATA1 splice mutation versus Control among ST-HSCs and MEPs). **d** Genomic DNA sequences upon CRISPR/Cas9 induced cleavage at the 5′ splice junction of exon 2 and their frequencies. Conserved sequence of wildtype 5′ exon splice site is highlighted in red (5′ end) and blue (3′ end). **e** Percentage of CRISPR/Cas9 efficiency as determined by single cell-derived colonies that were positive for STAG2 knock-out (only frame shift mutations). STAG2 is located on the X-chromosome and only male cord blood samples were utilized (n = 3 experiments with independent cord blood pools and >50 colonies per experiment). **f** Sanger sequencing analysis of single cell-derived colonies that were CRISPR/Cas9 edited for STAG2 knock-out. Example of a frame shift mutation is depicted. Error bars represent standard deviations.

Wagenblast et al. Supplementary Figure 7

a

NSG	LT-HSC dose	Transplantation engrafted/injected	Stem cell frequency
Control	25	2/10	1/104
	50	4/10	
	100	7/10	
	200	8/10	(Range: 65.5 - 164)

b

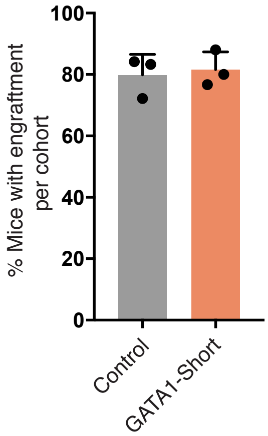

c

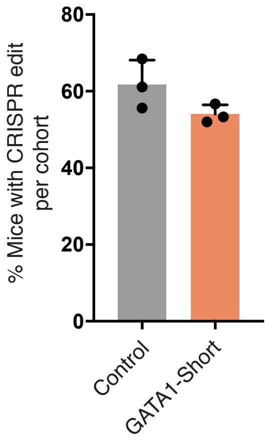

d

NSG	CRISPR/Cas9	mice injected	mice engrafted	engrafted mice with CRISPR edit
Cohort 1	Control	18	13	5
	GATA1-Short	25	22	10
Cohort 2	Control	19	16	10
	GATA1-Short	30	23	10
Cohort 3	Control	18	15	8
	GATA1-Short	30	24	10

e

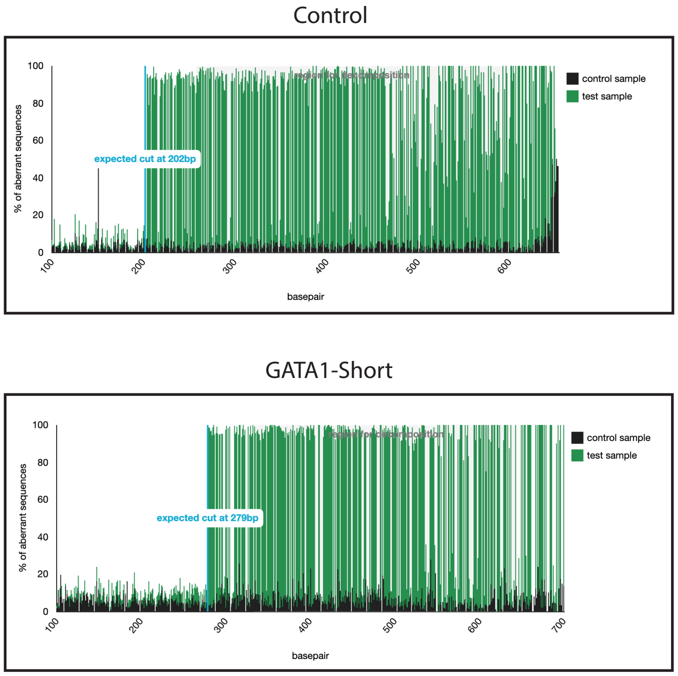

f

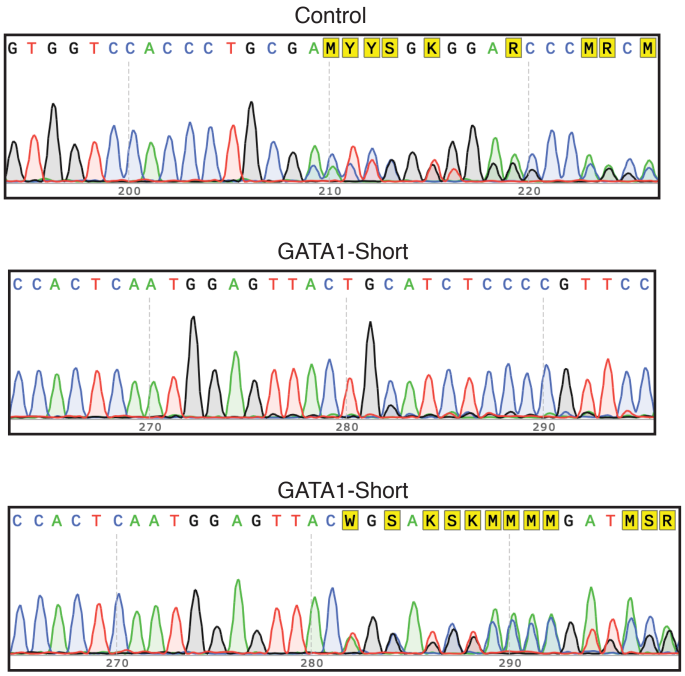

g

Mouse	Sequence of methylcellulose colonies (gRNA-1 to -2 junction)	Frequency
1	5'-TGGAACGGGGAGATGCAGTAACTCCATTGAGTGG-3'	24/24
2	5'-TGGAACGGGGAGATGTAACTCCATTGAGTGG-3'	20/20
3	5'-TGGAACGGGGAGATGCGTAACTCCATTGAGTGG-3'	22/22
4	5'-TGGAACGGGGAGATGCAGTAACTCCATTGAGTGG-3'	17/22
	5'-TGGAACGGGGAGATGCAACTCCATTGAGTGG-3'	5/22
5	5'-TGGAACGGGGAGGTAACTCCATTGAGTGG-3'	14/22
	5'-TGGAACGGGGAGATGCCACTCCATTGAGTGG-3'	8/22

Supplementary Figure 7: Setup of near-clonal xenotransplantation in NSG mice. a Limiting dilution analysis (LDA) of LT-HSCs transplanted NSG mice at varying doses for 20 weeks revealed a stem cell frequency of ~1/100 (n = 1 experiment). **b** Percentage of control and GATA1-Short edited LT-HSCs injected NSG mice with engraftment of >5% in the RF (based on human CD45+ expression) from each of three the cohorts (n = 3 experiments with independent cord blood pools). **c** Percentage of control and GATA1-Short edited LT-HSCs injected NSG mice with high CRISPR/Cas9 knock-out efficiency (>90% based on PCR and Sanger sequencing) from each of the three cohorts. **d** Number of mice utilized in each cohort and the number of mice that were used for analysis after validation of the CRISPR/Cas9 edit. **e** Analysis of chromatograms from cells of the RF of control and GATA1-Short edited LT-HSCs injected NSG mice. Percentage of aberrant sequencese at the gRNA cut site were utilized to assess CRISPR/Cas9 efficiency. **f** Examples of chromatograms from cells of the RF of control and GATA1-Short edited LT-HSCs injected NSG mice. Note the single clonal engraftment of a GATA1-Short edited LT-HSCs injected NSG mice based on the single chromatogram trace (second from top). **g** Sanger sequencing of individual colonies from methylcellulose colony formation using LT-HSC transplanted mice. No wildtype GATA1 colonies were detected (n = 5 mice). Error bars represent standard deviations.

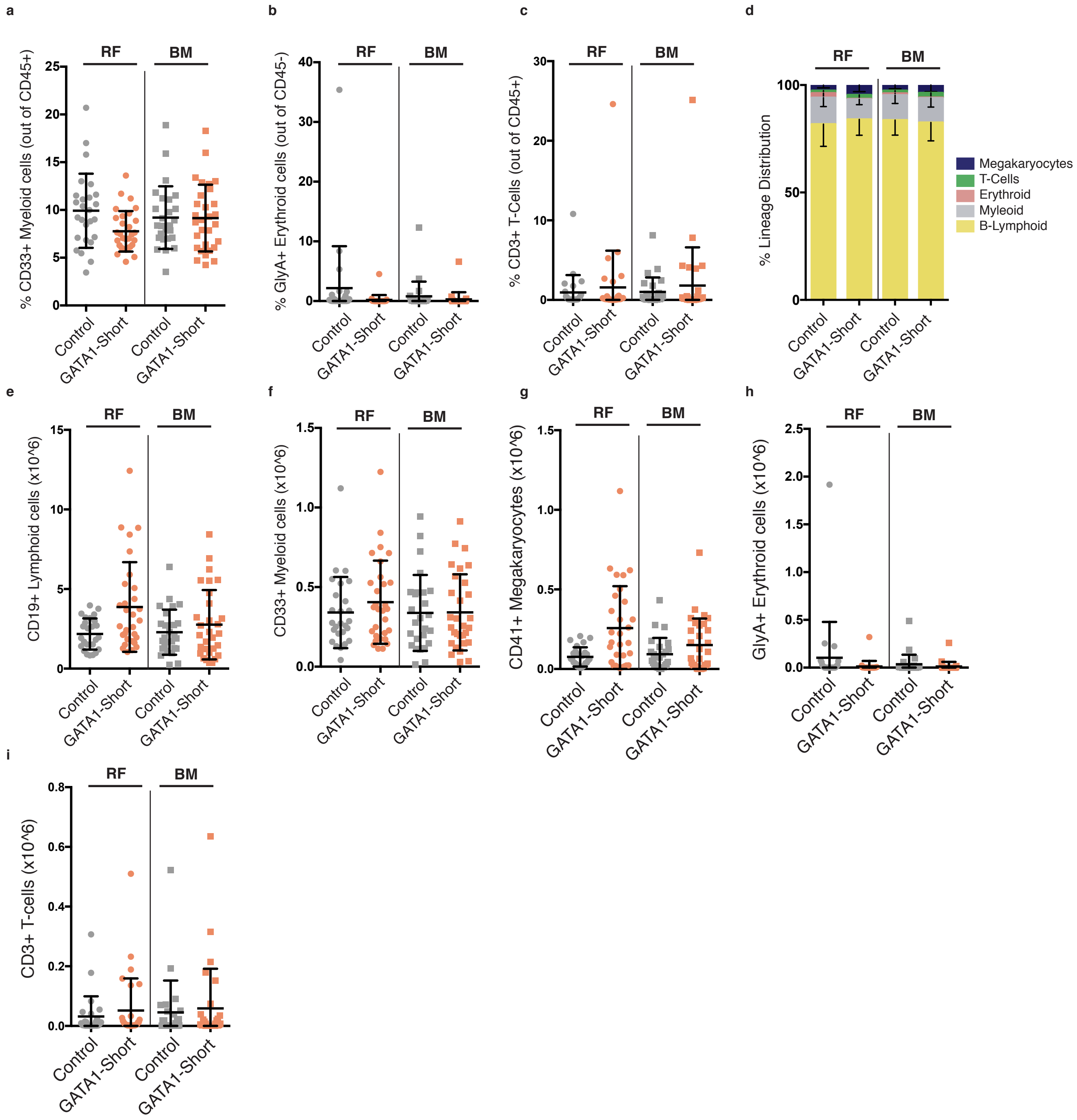

Supplementary Figure 8: Analysis of near-clonal xenotransplantation in NSG mice. a Percentage of CD33+CD45+ myeloid cells in control and GATA1-Short edited LT-HSCs injected NSG mice in RF and BM (n = 3 experiments with independent cord blood pools, unpaired t-test $P < 0.05$ for RF GATA1-Short versus RF control). **b** Percentage of GlyA+CD45– erythroid cells in control and GATA1-Short edited LT-HSCs injected NSG mice in the RF and BM. **c** Percentage of CD3+CD45+ T-cells in control and GATA1-Short edited LT-HSCs injected NSG mice in the RF and BM. **d** Percent distribution of all cell lineages in control and GATA1-Short edited LT-HSCs injected NSG mice in RF and BM. **e** Absolute cell numbers of CD19+CD45+ lymphoid cells in control and GATA1-Short edited LT-HSCs injected NSG mice in RF and BM (unpaired t-test $P < 0.01$ for RF GATA1-Short versus RF control). **f** Absolute cell numbers of CD33+CD45+ myeloid cells in control and GATA1-Short edited LT-HSCs injected NSG mice in RF and BM. **g** Absolute cell numbers of CD41+CD45– megakaryocytic cells in control and GATA1-Short edited LT-HSCs injected NSG mice in RF and BM (unpaired t-test $P < 0.005$ for RF GATA1-Short versus RF control). **h** Absolute cell numbers of GlyA+CD45– erythroid cells in control and GATA1-Short edited LT-HSCs injected NSG mice in RF and BM. **i** Absolute cell numbers of CD3+CD45+ T-cells in control and GATA1-Short edited LT-HSCs injected NSG mice in RF and BM. Error bars represent standard deviations.

a

NSGW41	LT-HSC dose	Transplantation engrafted/injected	Stem cell frequency
Control	25	2/10	1/175
	50	2/10	
	100	4/10	(Range: 104 - 295)
	200	7/10	

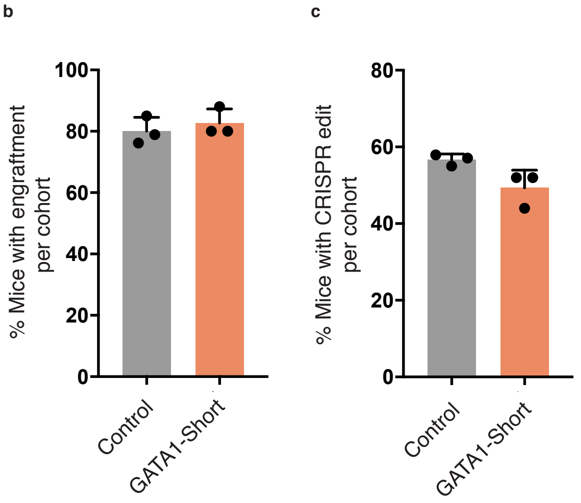

d

NSGW41	CRISPR/Cas9	mice injected	mice engrafted	engrafted mice with CRISPR edit
Cohort 1	Control	19	15	7
	GATA1-Short	25	22	10
Cohort 2	Control	21	16	7
	GATA1-Short	25	20	8
Cohort 3	Control	20	17	8
	GATA1-Short	25	20	6

Supplementary Figure 9: Setup of near clonal xenotransplantation in NSGW41 mice. a Limiting dilution analysis (LDA) of LT-HSCs transplanted NSGW41 mice at varying doses for 12 weeks revealed a stem cell frequency of ~1/175 (n = 1 experiment). **b** Percentage of control and GATA1-Short edited LT-HSCs injected NSGW41 mice with engraftment of >5% in the RF (based on human CD45+ expression, n = 3 experiments with independent cord blood pools). **c** Percentage of control and GATA1-Short edited LT-HSCs injected NSGW41 mice with high CRISPR/Cas9 knock-out efficiency (>90% based on PCR and Sanger sequencing). **d** Number of mice utilized in each cohort and the number of mice that were used for analysis after validation of the CRISPR/Cas9 edit. Error bars represent standard deviations.

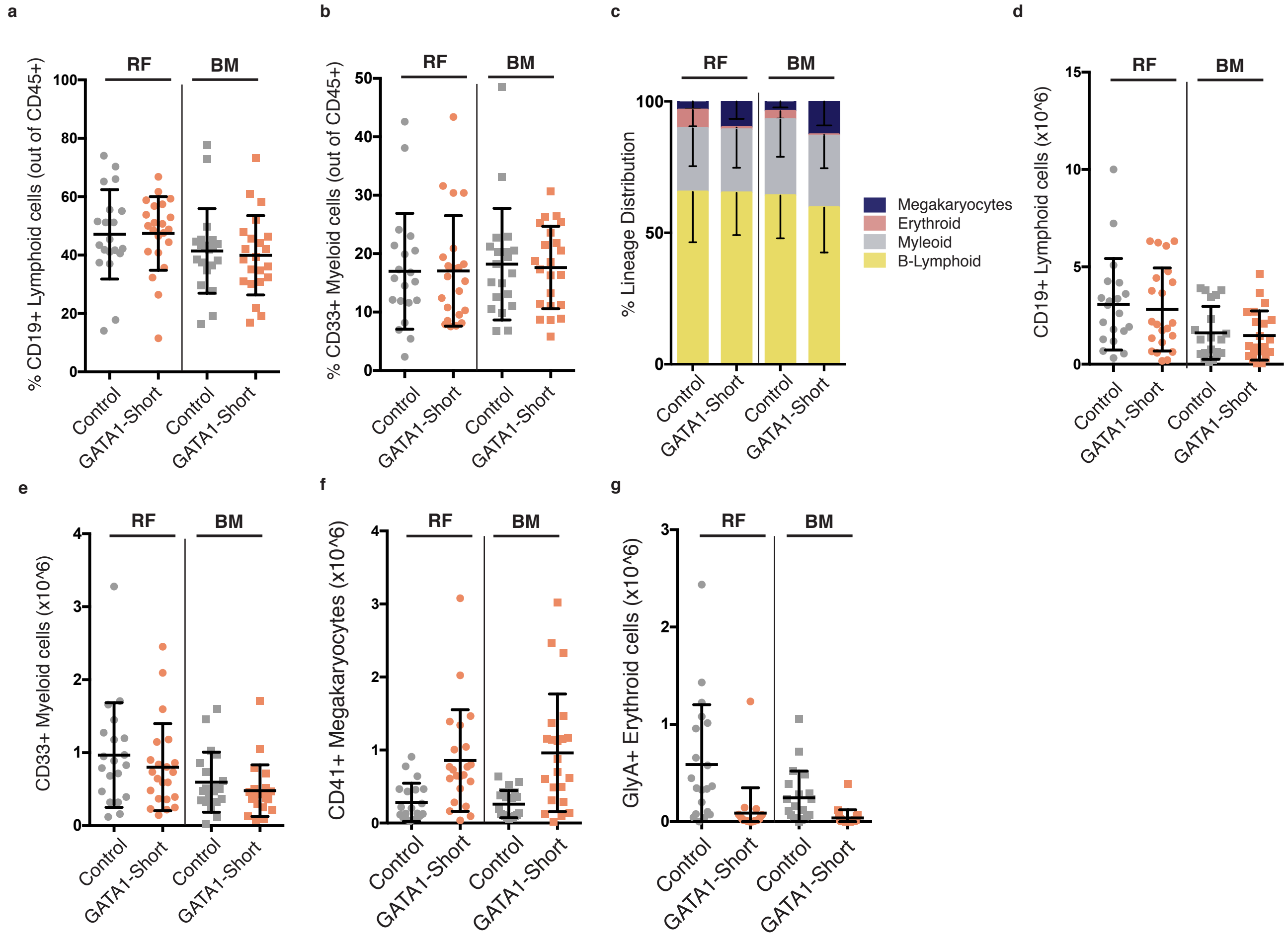

Supplementary Figure 10: Analysis of near clonal xenotransplantation in NSGW41 mice. a Percentage of CD19+CD45+ lymphoid cells in control and GATA1-Short edited LT-HSCs injected NSGW41 mice in RF and BM (n = 3 experiments with independent cord blood pools). **b** Percentage of CD33+CD45+ myeloid cells in control and GATA1-Short edited LT-HSCs injected NSGW41 mice in RF and BM. **c** Percent distribution of all cell lineages in control and GATA1-Short edited LT-HSCs injected NSGW41 mice in RF and BM. **d** Absolute cell numbers of CD19+CD45+ lymphoid cells in control and GATA1-Short edited LT-HSCs injected NSGW41 mice in RF and BM. **e** Absolute cell numbers of CD33+CD45+ myeloid cells in control and GATA1-Short edited LT-HSCs injected NSGW41 mice in RF and BM. **f** Absolute cell numbers of CD41+CD45– megakaryocytes in control and GATA1-Short edited LT-HSCs injected NSGW41 mice in RF and BM (unpaired t-test $P < 0.005$ for GATA1-Short versus control for both RF and BM). **g** Absolute cell numbers of GlyA+CD45– erythroid cells in control and GATA1-Short edited LT-HSCs injected NSGW41 mice in RF and BM (unpaired t-test $P < 0.005$ for GATA1-Short versus control in both RF and BM). Error bars represent standard deviations.

Wagenblast et al. Supplementary Figure 11

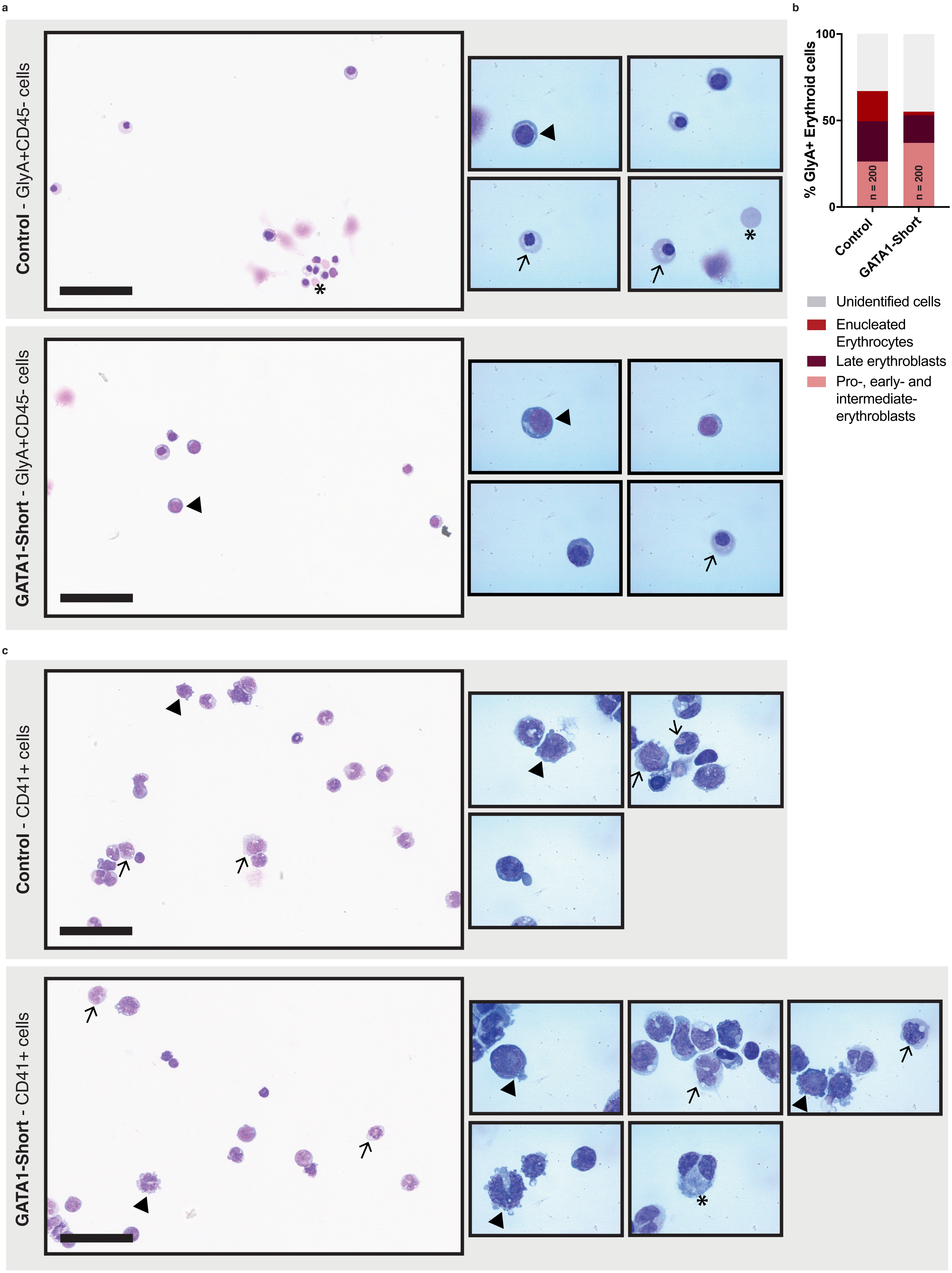

Supplementary Figure 11: Morphological analysis of sorted erythroid and megakaryocytic populations. a Cytomorphology of GlyA+CD45– sorted cells from control and GATA1-Short edited LT-HSCs injected NSGW41 mice from the RF. Cells were cytospinned and stained with Giemsa. Erythroid precursors (arrowhead), such as pro-, early- and intermediate-erythroblasts, late erythroblasts (arrow) and enucleated erythrocytes (asterisk) are seen. More immature forms of erythroid cells and fewer enucleated erythrocytes are visible in GATA1-Short edited LT-HSCs injected NSGW41 mice compared to control. (n = 5 mice pooled together, scale is 60 um) **b** Quantification of **a** (n = 200 cells). **c** Cytomorphology of CD41+ sorted cells from control and GATA1-Short edited LT-HSCs injected NSGW41 mice from the BM. Cells with variable amounts of blue-greyish cytoplasm, sometimes with a few vacuoles, and with fine granules and pleomorphic nuclei (arrow) were frequently seen. Cells with cytoplasmatic pseudopod formation, sometimes exhibiting distinct nucleoli (arrowhead) were also observed with less frequency. Finally, few large binucleated cells were observed (asterisk). No striking differences in morphology were observed between CD41+ cells from GATA1-Short edited LT-HSCs injected NSGW41 mice compared to control. Sufficient numbers of CD41+CD45- cells could not be detected after the freeze/thaw cycle of stored BM and thus only bulk CD41+ cells were sorted, regardless of CD45 staining. (n = 5 mice pooled together, scale is 60 um).

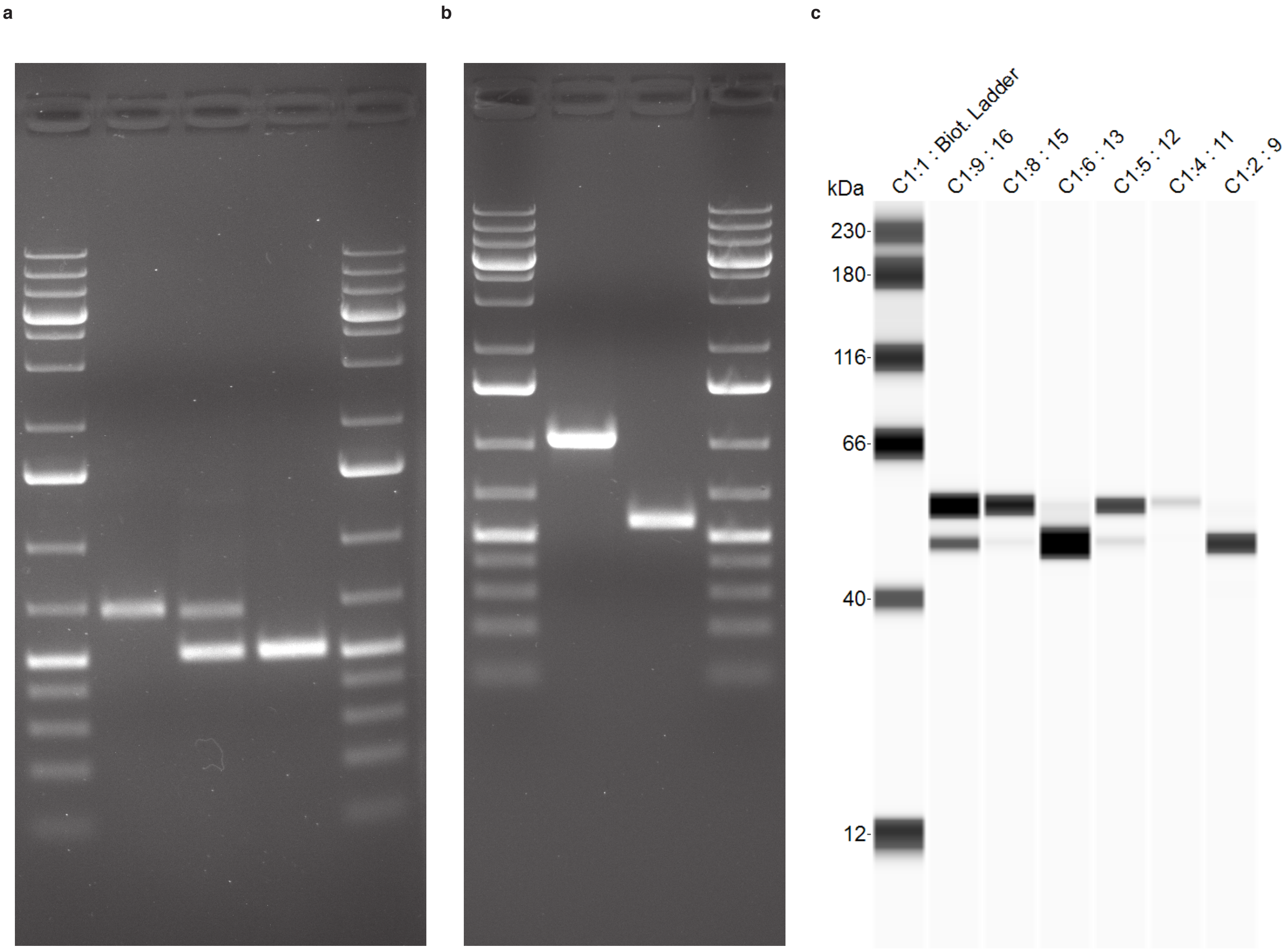

Supplementary Figure 12: Full length gel pictures and western assay. a Gel electrophoresis picture from Supplementary Fig. 4a. **b** Gel electrophoresis picture from Supplementary Fig. 4b. **c** Western assay image from Fig. 2c and Supplementary Fig. 6a.
