# Supplementary material for: Functional profiling of single CRISPR/Cas9-edited human long-term hematopoietic stem cells
Source: Nat Commun. 2019 Oct 18;10:4730. doi: 10.1038/s41467-019-12726-0 (PMC6802205; doi:10.1038/s41467-019-12726-0)
Supplement: Supplementary file 3 — Reporting Summary [file 41467_2019_12726_MOESM3_ESM.pdf]

nature research

Corresponding author(s): John E. Dick & Eric R. Lechman

Last updated by author(s): Sep 2, 2019

Reporting Summary

Nature Research wishes to improve the reproducibility of the work that we publish. This form provides structure for consistency and transparency in reporting. For further information on Nature Research policies, see Authors & Referees and the Editorial Policy Checklist.

Statistics

For all statistical analyses, confirm that the following items are present in the figure legend, table legend, main text, or Methods section.

n/a	Confirmed	
☐	☒	The exact sample size (*n*) for each experimental group/condition, given as a discrete number and unit of measurement
☐	☒	A statement on whether measurements were taken from distinct samples or whether the same sample was measured repeatedly
☐	☒	The statistical test(s) used AND whether they are one- or two-sided<br>*Only common tests should be described solely by name; describe more complex techniques in the Methods section.*
☒	☐	A description of all covariates tested
☒	☐	A description of any assumptions or corrections, such as tests of normality and adjustment for multiple comparisons
☒	☐	A full description of the statistical parameters including central tendency (e.g. means) or other basic estimates (e.g. regression coefficient) AND variation (e.g. standard deviation) or associated estimates of uncertainty (e.g. confidence intervals)
☒	☐	For null hypothesis testing, the test statistic (e.g. *F*, *t*, *r*) with confidence intervals, effect sizes, degrees of freedom and *P* value noted<br>*Give P values as exact values whenever suitable.*
☒	☐	For Bayesian analysis, information on the choice of priors and Markov chain Monte Carlo settings
☒	☐	For hierarchical and complex designs, identification of the appropriate level for tests and full reporting of outcomes
☒	☐	Estimates of effect sizes (e.g. Cohen's *d*, Pearson's *r*), indicating how they were calculated

Our web collection on statistics for biologists contains articles on many of the points above.

Software and code

Policy information about availability of computer code

Data collection	FACSDiva 8.0
Data analysis	FlowJo 10.4, Prism 7.0, Tide 2.0.1, Benchling, Cas0-OFFinder

For manuscripts utilizing custom algorithms or software that are central to the research but not yet described in published literature, software must be made available to editors/reviewers. We strongly encourage code deposition in a community repository (e.g. GitHub). See the Nature Research guidelines for submitting code & software for further information.

Data

Policy information about availability of data

All manuscripts must include a data availability statement. This statement should provide the following information, where applicable:

- Accession codes, unique identifiers, or web links for publicly available datasets
- A list of figures that have associated raw data
- A description of any restrictions on data availability

All datasets generated in this study are available within the paper or from the corresponding author upon reasonable request.

Field-specific reporting

Please select the one below that is the best fit for your research. If you are not sure, read the appropriate sections before making your selection.

☒ Life sciences ☐ Behavioural & social sciences ☐ Ecological, evolutionary & environmental sciences

For a reference copy of the document with all sections, see nature.com/documents/nr-reporting-summary-flat.pdf

Life sciences study design

All studies must disclose on these points even when the disclosure is negative.

Sample size	Sample size was chosen to give sufficient power for calling significance with standard statistical tests. In vitro single cell differentiation assays and in vivo xenotransplantations were were performed with three independent cord blood pools.
Data exclusions	No data points were excluded.
Replication	All experiments have been performed three times in independent cord blood pools.
Randomization	In vitro single cell differentiation assays were split into different groups based on 96 well plates. In vivo groups were based on cage location.
Blinding	All flow cytometry quantification was performed in a blinded manner.

Reporting for specific materials, systems and methods

We require information from authors about some types of materials, experimental systems and methods used in many studies. Here, indicate whether each material, system or method listed is relevant to your study. If you are not sure if a list item applies to your research, read the appropriate section before selecting a response.

Materials & experimental systems

n/a	Involved in the study	
☐	☒	Antibodies
☒	☐	Eukaryotic cell lines
☒	☐	Palaeontology
☐	☒	Animals and other organisms
☐	☒	Human research participants
☒	☐	Clinical data

Methods

n/a	Involved in the study	
☒	☐	ChIP-seq
☐	☒	Flow cytometry
☒	☐	MRI-based neuroimaging

Antibodies

Antibodies used	All antibodies from BD Biosciences, unless stated otherwise: CD45RA FITC (555488, HI100), CD49f PE-Cy5 (551129, GoH3), CD10 BV421 (562902, HI10a), CD19 V450 (560353, HIB19), FLT3 CD135 biotin (clone 4G8, custom conjugation), CD45 V500 (560777, HI30), CD34 APC-Cy7 (clone 581, custom conjugation), CD38 PE-Cy7 (335825, HB7), CD90 APC (559869, 5E10), CD7 A700 (561603, M-T701), Streptavidin Conjugate Qdot 605 (ThermoFisher, Q10101MP), CD45 APC (560777, HI30), CD34 APC-Cy7 (clone 581, custom conjugation), CD33 BV421 (Biolegend, 303416, WM53), CD71 FITC (347513, L01.1), CD41 PE-Cy5 (Beckman Coulter, 6607116, P2) and GlyA PE (Beckman Coulter, IM2211U, KC16)
Validation	All antibodies were validated in previous publications from our lab.

Animals and other organisms

Policy information about studies involving animals; ARRIVE guidelines recommended for reporting animal research

Laboratory animals	All mouse transplants were performed with 8- to 12-week-old female NOD.Cg-PrkdcscidIl2rgtm1Wjl/SzJ (NSG) mice (JAX) that were sublethally irradiated with 225cGy, 24 hours before transplantation, or with 8- to 12-week-old female NOD.Cg-PrkdcscidIl2rgtm1WjlKitem1Mvw/SzJ (NSGW41) mice (kind gift from Lenny Schultz) that were not irradiated.
Wild animals	NA
Field-collected samples	NA
Ethics oversight	All mouse experiments were approved by the University Health Network (UHN) Animal Care Committee.

Note that full information on the approval of the study protocol must also be provided in the manuscript.

Human research participants

Policy information about studies involving human research participants

Population characteristics	Male cord blood samples were utilized.
Recruitment	Human cord blood samples were obtained from Trillium and William Osler hospitals. There were no selection biases.

Ethics oversight	Human cord blood samples were obtained with informed consent in accordance to guidelines approved by University Health Network (UHN) Research Ethics Board.

Note that full information on the approval of the study protocol must also be provided in the manuscript.

Flow Cytometry

Plots

Confirm that:

☒ The axis labels state the marker and fluorochrome used (e.g. CD4-FITC).

☒ The axis scales are clearly visible. Include numbers along axes only for bottom left plot of group (a 'group' is an analysis of identical markers).

☒ All plots are contour plots with outliers or pseudocolor plots.

☒ A numerical value for number of cells or percentage (with statistics) is provided.

Methodology

Sample preparation	Biological source and sample preparation are described in the manuscript. In short, human cord blood samples were lineage depleted and purified for LT-HSCs, ST-HSCs and MEPs using flow cytometry.
Instrument	Cell sorting was performed on the FACSAria III (BD Biosciences).
Software	FACSDiva 8.0 was used to collect data. FlowJo 10.4 was utilized to analyze data.
Cell population abundance	Post-sort fractions were >95% as determined by a post-sort purity check.
Gating strategy	The gating strategy is described in the manuscript.

☒ Tick this box to confirm that a figure exemplifying the gating strategy is provided in the Supplementary Information.
